# Effects of Hydrogen Sulfide on Bacterial Communities on the Surface of Galatheid Crab, *Shinkaia crosnieri*, and in a Bacterial Mat Cultured in Rearing Tanks

**DOI:** 10.1264/jsme2.ME12070

**Published:** 2012-10-19

**Authors:** Masaaki Konishi, Tomo-o Watsuji, Satoshi Nakagawa, Yuji Hatada, Ken Takai, Takashi Toyofuku

**Affiliations:** 1 Institute of Biogeoscience (Biogeos), Japan Agency for Marine-Earth Science and Technology (JAMSTEC), 2–15 Natsushima-cho, Yokosuka, Kanagawa 237–0061, Japan; 2 Faculty of Fisheries Sciences, Hokkaido University, 3–1–1 Minato-cho, Hakodate, Hokkaido 041–8611, Japan

**Keywords:** hydrogen sulfide, epibiont, symbiont, tank, feedback control

## Abstract

To investigate the effects of H_2_S on the bacterial consortia on the galatheid crab, *Shinkaia crosnieri*, crabs of this species were cultivated in the laboratory under two different conditions, with and without hydrogen sulfide feeding. We developed a novel rearing tank system equipped with a feedback controller using a semiconductor sensor for hydrogen sulfide feeding. H_2_S aqueous concentration was successfully maintained between 5 to 40 μM for 80 d with the exception of brief periods of mechanical issues. According to real-time PCR analysis, the numbers of copies of partial 16S rRNA gene of an episymbiont of the crabs with H_2_S feeding was three orders of magnitude larger than that without feeding. By phylogenetic analysis of partial 16S rRNA gene, we detected several clones related to symbionts of deep sea organisms in *Alphaproteobacteria*, *Gammaproteobacteria*, *Epsilonproteobacteria*, and *Flavobacteria*, from a crab with H_2_S feeding. The symbiont-related clones were grouped into four different groups: *Gammaproteobacteria* in marine epibiont group I, *Sulfurovum*-affiliated *Epsilonproteobacteria*, *Osedax mucofloris* endosymbiont-affiliated *Epsilonproteobacteria*, and *Flavobacteria* closely related to CFB group bacterial epibiont of *Rimicaris exoculata*. The other phylotypes were related to *Roseobacter*, and some *Flavobacteria*, seemed to be free-living psychrophiles. Furthermore, white biofilm occurred on the surface of the rearing tank with H_2_S feeding. The biofilms contained various phylotypes of *Gammaproteobacteria*, *Epsilonproteobacteria*, and *Flavobacteria*, as determined by phylogenetic analysis. Interestingly, major clones were related to symbionts of *Alviniconcha* sp. type 2 and to endosymbionts of *Osedax mucofloris*, in *Epsilonproteobacteria*.

The chemoautotrophic ecosystem is supported by chemoautotrophs, which are organisms that are capable of fixing carbon using chemical energy obtained from the oxidation of reduced compounds blowing up from hydrothermal vents, such as sulfuric compounds, methane, and hydrogen (18). Several benthic invertebrates are hosts of chemoautotrophic bacteria in cells of specialized tissues and on the surface of their bodies, and they obtain nutrition from the bacteria (2).

*Shinkaia crosnieri*, a galatheid crab, is one of the most predominant animal species that massively aggregate close to hydrothermal vents on the subseafloor in the deep sea. The crab is observed only in areas near active vents in the Okinawa Trough, western Pacific (25, 26). The crab has not only strong, stout, sparse setae on the dorsal surface of its carapace, chelipeds, and walking legs, but also long, soft, dense plumose setae on the ventral surface of its body (1). According to previous studies (27, 30), epibiotic filamentous symbionts inhabit the surface of the plumose. Phylogenetic analyses of 16S rRNA sequences in those studies showed no significant difference in the epibiotic phylotype composition of the individuals collected from different sites. The major subdivisions of the phylotypes were *Epsilonproteobacteria* and *Gammaproteobacteria*. In particular, specific phylogenetic clusters related to the genus *Sulfurovum* have been found in epibiotic 16S rRNA gene clone libraries. A free-living *Sulfurovum* sp. NBC37-1 isolated from a vent field is one of the most well-characterized strains (17, 31). According to whole genome sequencing (17), NBC37-1 is a mesophilic hydrogen- and sulfur-oxidizing chemolithoautotoroph and has key genes associated with sulfur reduction (*psr* families) and oxidation (*sox* families). Further culture-dependent experiments showed that the strain can grow utilizing two different types of sulfur related energy metabolism, namely, hydrogen-oxidizing sulfur respiration and thiosulfate-oxidizing nitrate/oxygen respiration in the deep sea (17). Such experiments also provide evidence for the operation of the hydrogenase-coupled polysulfide reductase pathway as well as the Sox system; therefore, the strain and its relatives may inhabit the mixing zones between oxidative and reductive areas where the physical and chemical conditions are optimal for sulfur-oxidizing nitrate/oxygen and hydrogen-oxidizing respiration (31).

On the other hand, Saito (21) reported that *Shinkaia crosnieri* obtains many of its lipids from *Bathymodiolus* mussels and chemosynthetic symbionts on the basis of the characteristics of fatty acid composition of the crab. Miyake *et al.* (15) have reported that the crabs graze on epibiotic bacteria using their mouth-parts in a tank rearing system with an artificial hydrothermal vent system; therefore, we consider that the crab is able to obtain many nutrients from episymbionts on its surface. However, there has been no information on how environmental, physical and chemical conditions affect the episymbiont consortium on the surface of the crab, since it is difficult to control experimental conditions, particularly H_2_S concentration, in the laboratory. The continuous supply of hydrogen sulfide, which is a highly poisonous and reactive gas, at low levels has also been difficult.

In the applied microbiology research field, there have been reported high cell density cultivation techniques with feedback control of volatile and toxic materials such as alcohols at a low level in a jar fermenter by detecting evaporated compounds in the exhaust gas by a semiconductor sensor (6, 9). We applied technology for the H_2_S control system of the rearing tank for deep-sea invertebrates, which is also capable of controlling temperature and dissolved oxygen. Using the newly developed rearing tank system, we first examined the effect of low-concentration H_2_S on episymbiotic bacterial communities in an artificial environment. The aim of this experiment was to examine the effects in clear cause-effect relationships between H_2_S and bacterial communities by novel controlled growth conditions in the rearing tanks. Moreover, a white bacterial mat was obtained from the surface of the rearing tank. On the basis of the results of phylogenetic analysis, we also describe here the bacterial consortium structure. Finally, we discuss the effects of hydrogen sulfide on deep-sea bacteria and the potential use of the rearing tank experimental system.

## Materials and Methods

### Collection of *S. crosnieri* from deep-sea vent

Individuals of *S. crosnieri* were collected from a deep-sea hydrothermal field: Iheya North field (27°47.46′N, 126°53.80′E, depth = 981 m) in the Okinawa Trough, Japan, by a remotely operated vehicle (ROV), ‘*HyperDolphin*’ of Japan Agency for Marine-Earth Science and Technology (JAMSTEC). The individuals collected, which were still alive after the rapid pressure and temperature change during recovery, were immediately moved to shipboard tanks containing surface seawater at 4°C.

### Rearing tanks for *S. crosnieri*

**Fig. 1:**
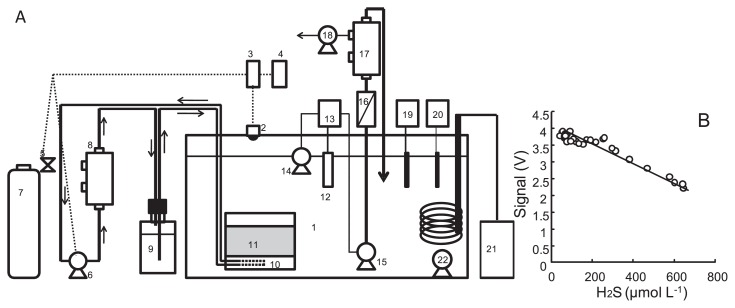
Schematic illustration of experimental setup, A. 1: rearing tank, 2: gas sensor unit, 3: detect relay, 4: data logger, 5: electromagnetic valve, 6: pump, 7: cylinder of liquid hydrogen sulfide, 8: gas-exchange unit, 9: gas trap, 10: porous PTFE membrane tube, 10: bed of sea sand, 12: DO sensor, 13: DO terminator, 14: aeration pump, 15: pump, 16: EHEIM filter, 17: degassing unit, 18: degassing pump, 19: pH meter, 20: thermostat, 21: cooling unit, and 22: pump. Plots of gas sensor signals versus hydrogen sulfide concentrations in tank water, B.

Fig. 1A shows the rearing tank system including the H_2_S feeding system (gas sensor, feedback control system, and gas-providing apparatuses), temperature control system (a thermostat controller and a cooler), and dissolved oxygen (DO) control system (DO sensor, mixing pump, and degassing system). The main tank of 100 and 200 L and their lids (1 in Fig. 1A) were made of acryl plastic plates from Iwaki Pumps (Tokyo, Japan). The tin dioxide semiconductor sensor unit for H_2_S (2 in Fig. 1A, TGS-825; Figaro Eng., Osaka, Japan) was equipped with an electric circuit unit placed inside the lid of the rearing tank. The sensor detected H_2_S in the head space of the tank as voltage signals. The signals are approximately proportional to the H_2_S concentration in tank water (Fig. 1B). The sensor was connected to a detection relay (3 in Fig. 1A, 2411D-1-04-H0-T5-A; Tsuruga Electric, Osaka, Japan) and a data logger (4 in Fig. 1A, LR5042; Hioki, Nagano, Japan). An electromagnetic valve and a pump were controlled by on-off signals by the detect relay. Hydrogen sulfide gas was provided from the cylinder of liquefied H_2_S via the valve to a gas exchange unit (8 in Fig. 1A, STERAPORE degassing module, MHF0504MBFT; Mitsubishi Rayon, Tokyo, Japan). The gas was dissolved in water that passed through the unit. The water was degassed by a gas trap (9 in Fig. 1A) and introduced into a porous tetrafluoroethylene (PTFE) tube (10 in Fig. 1A, POREFLON tube, TB-0302; Sumitomo Electric Fine Polymer, Tokyo, Japan), and circulated to the gas-exchange unit. The dissolved H_2_S was diffused from the circulating water to the tank water via the PTFE tube and sea sand bed (11 in Fig. 1A). The H_2_S concentration in the tank water was controlled to between 20 and 50 μmol L^−1^. The concentration was set to avoid chemical generation of white matter in the tank water by H_2_S in preparatory experiments, because the optimal concentration for the crabs was unknown. The concentration of H_2_S in the Iheya North field has been reported to be over a wide range from 2.6 mmol kg^−1^ to not detected (7). A digital optical sensor (Oxymax W COS61-D; Endress+Hauser, Switzerland) was used for detecting DO. The signals were analyzed by a transmitter (Liquisis M COM223; Endress+Hauser). The aeration pump (14 in Fig. 1A) and degassing system (16, 17, and 18 in Fig. 1A) were controlled by the transmitter. DO was controlled by the system at between 1.0 and 1.5 mg L^−1^, which corresponds to between 8.12 and 12.2% of saturation at 5.2°C. pH was continuously measured by a pH sensor (MicropH; Aquabase, Kanagawa, Japan). Temperature was controlled at 5.25±2.5°C by a thermostat controller (TC-100; Iwaki Pumps) and a cooling unit (AZ-151X; Iwaki Pumps). Artificial seawater (ROHTOMARINE II; Iwaki Pumps) was used as the tank water. For a comparative experiment without H_2_S feeding, a tank without an H_2_S feeding system (2 to 11 in Fig. 1A) was also prepared. Sixty crabs were transferred to 200-L tanks with an H_2_S feeding system, and thirty crabs to a 100-L tank without a feeding system.

### Determination of H_2_S concentration in tank water

A colorimetric method for the quantification of H_2_S concentration in tank water was carried out by a previously reported method (32) with modification. *N,N*-dimethyl-*p*-phenylene diammonium dichloride (14.3 mM) and FeCl_3_ (18.5 mM), solutions were prepared in 0.6 M HCl as test reagents. One milliliter of tank water was gently mixed with 0.1 mL of a test reagent. The samples were incubated at room temperature for 5 min. The absorbance of the samples at 670 nm was measured by a spectrometer (UV-1000; Hitachi, Tokyo, Japan).

### Microscopy

Microscopy was carried out using microscope systems (Eclipse E600, Nikon, and Olympus BX51 systems) equipped with a digital camera. Then, 4′,6-diamidine-2′-phenylindole dihydrochloride (DAPI)-stained samples were prepared as follows. Samples of the setae of *S. crosnieri* crabs and biofilms were collected in deionized water and centrifuged at 10,000×*g* for 1 min. The supernatant was removed and the samples were washed in 1 μg mL^−1^ DAPI in methanol solution and covered for 15 min at room temperature with DAPI solution. DAPI solution was removed by centrifugation, and the setae were then gently washed with methanol immediately. The washed samples were redispersed in phosphate-buffered saline (pH 6.8) and used for microscopy.

### DNA extraction

Samples of the setae of *S. crosnieri* (approximately 20 mg wet weight) were collected immediately after washing the crabs with filtered artificial sea water, and centrifuged at 9,100×*g* for 1 min. The supernatant was removed carefully. Wet weight of a portion of setae samples was measured. Genomic DNA of samples were extracted from setae immediately by a DNA extraction kit for soil (ISOIL for Beads Beating; Nippon Gene, Tokyo, Japan). Extraction was carried out following the manufacturer’s instructions.

### Real-time PCR analysis

To estimate the population of bacteria on the setae of the crabs, which were cultivated with and without feeding with H_2_S, real-time PCR was performed. A SYBR Premix Ex Taq II kit (Takara Bio, Shiga, Japan) was used for real-time PCR analysis. Partial 16S rRNA gene sequences as target genes were amplified by PCR using the SYBR Premix Ex Taq II system with the universal primers Bac341F (3′-CCTACGGGAGGCAGCAGTGA-5′) and Bac534R (3′-CGTATTACCGCGGCTGCTG-5′). Thermal cycling and light detection were performed using the ABI Prism 7500 system (Applied Biosystems, Foster City, CA, USA), with preliminary denaturation at 94°C for 4 min, followed by 40 cycles of denaturation at 94°C for 30 s, annealing at 55°C for 30 s, and elongation at 72°C for 34 s, and following the melt curve stage, denaturation at 95°C for 15 s, annealing at 60°C for 1 min, and denaturation at 95°C for 15 s. Standard DNA solutions (1.2×10^3^ to 1.2×10^9^ copies μL^−1^) was prepared from genomic DNA of *Escherichia coli* HB101 by PCR amplification using ABI GeneAmp PCR System 2700 (Applied Biosystems) with Bac341F and Bac534R primers. A Qubit Assay kit and a Qubit fluorometer were used for the quantification of standard DNA. Quantification was carried out in accordance with the manufacturers’ instructions. The amount of bacteria against wet weight was calculated as the number of copies per gram from *Ct* of individual reaction curves using the standard program of ABI Prism 7500. Real-time PCR analysis was performed three times for three individuals.

### Phylogenetic analysis

To analyze epibiotic clones on the surface of the crabs, the obtained real-time PCR products were used directly as follows. The products were purified using the Wizard SV Gel and PCR CleanUp System (Promega Corporation, Madison, WI, USA). Purified products were cloned using a TA cloning kit (QIAGEN PCR Cloning*^plus^* kit; QIAGEN, Germany) in accordance with the manufacturer’s instructions. The recombinant colonies were transferred to LB medium with 50 μg mL^−1^ ampicillin and incubated at 37°C overnight. Recombinant plasmids were extracted using the QIAprep Miniprep system (QIAGEN) and sequenced with an ABI PRISM 3100 DNA Analyzer and an BigDye Terminator Cycle Sequencing kit, version 3.1 (PE Biosystems, Foster City, CA, USA). In the analysis of bacterial biofilms, 16S rRNA gene sequences were amplified with the bacterial 16S rRNA-universal oligonucleotide primers Bac27F and Univ1492R (11). Thermal cycling was performed using a GeneAmp PCR System 2700 (Applied Biosystems), with preliminary denaturation at 94°C for 4 min, followed by 27 cycles of denaturation at 94°C for 30 s, annealing at 55°C for 30 s, and elongation at 72°C for 2 min, and a final elongation at 72°C for 7 min. The amplified 16S rRNA gene products were cloned using the above TA cloning kit. The TA clones were used for sequencing and phylogenetic analysis. Single phylogenetic clone types (phylotypes) were obtained from the sequence analyses. The phylotypes were compared with those of related sequences obtained from DNA Data Bank, Japan (DDBJ) and GenBank of the National Center for Biotechnology Information via the BLAST search program and aligned using Clustal X ver. 2.0 software by the neighbor-joining method (12). The phylogenetic tree was visualized using TreeView software version 1.6.6.

### Nucleotide sequence accession numbers

The sequence data have been submitted to EMBL/GenBank/DDBJ databases under accession numbers AB697010 to AB697038.

## Results

### Cultivation of *S. crosnieri* with epibionts

**Fig. 2:**
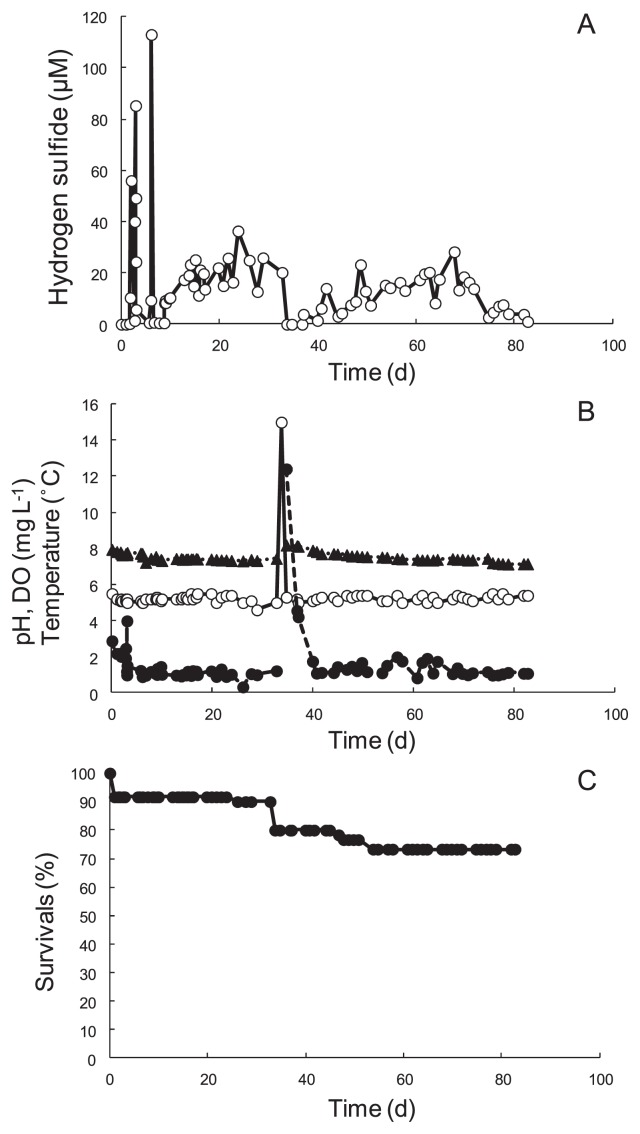
Time courses of H_2_S concentration, pH, DO concentration, temperatures, and survival rate. Time courses of H_2_S concentration (A), pH, DO, and temperature (B), and survival rate of the crab as a percentage (C). Symbols: pH (▲), DO (●), and temperatures (○).

**Fig. 3:**
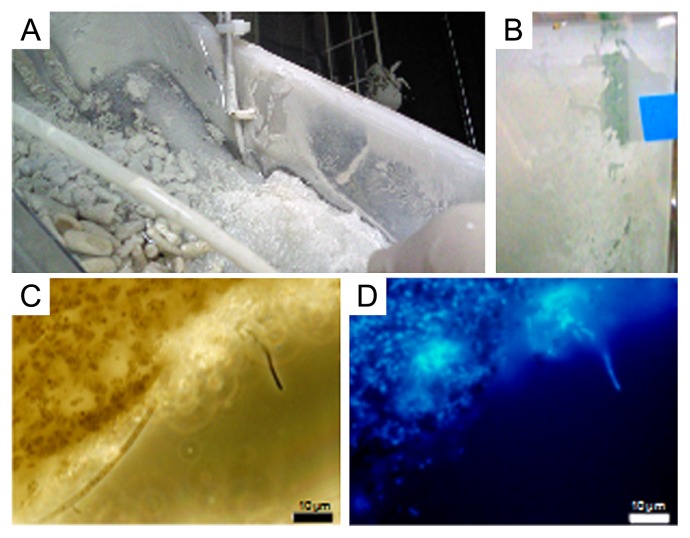
Images of biofilms. Image of biofilm occurring on the surface of the tank wall (A), image of biofilm occurring on the surface of the sea sand bed (B, 11 in Fig. 1A), phase difference microscopy image of a portion of biofilm (C), and epifluorescence microscopy image of a portion of DAPI-stained biofilm (D).

To investigate the effects of H_2_S feeding that are separate from the other effects, including hydrostatic pressure, temperature, and the other chemicals as far as possible, *S. crosnieri* crabs were cultivated in rearing tanks with and without H_2_S feeding. The temperature and DO of the rearing tank without H_2_S feeding were maintained at 5°C and 1 mg L^−1^ as set points during the cultivation period. pH was kept at approximately 8.0 during cultivation periods (data not shown). Fig. 2 indicates the parameters measured during the cultivation with H_2_S feeding. Four crabs died 1 d after starting cultivation when H_2_S was not fed into the tank; stress during shipping probably affected these dead crabs. In the early phase of cultivation (first week), the effects of H_2_S on the activities of the crabs were examined. H_2_S was initially introduced in pulses until an H_2_S concentration of approximately 100 μmol L^−1^ was reached in the tank water (Fig. 2A). The concentration of this compound increased rapidly during feeding and decreased markedly after stopping the feeding. The rapid decrease in H_2_S concentrations may have mainly been due to the spontaneous oxidization of H_2_S. The initial pulse introduction of the compound did not have harmful effects on the survival of the crabs (Fig. 2C). Then, H_2_S was continuously introduced into the tank, and its concentration was maintained between 5 and 60 μmol L^−1^ by the feedback control system. Feeding was successfully maintained before the cooling unit malfunctioned 36 d after starting the cultivation. During the period before the malfunction, the temperature of the tank water was well controlled at 5.2±0.2°C, and DO concentration initially decreased to approximately 1 mg L^−1^ in the initial 7 d (Fig. 2B). The decrease in DO concentration may have been caused by not only the degassing-DO control system, but also the respiration of crabs and aerobic bacterium. pH gradually decreased from 7.95 to 7.45 during the same period (Fig. 2B). Unfortunately, the malfunction of the cooling unit markedly increased the tank temperature to 12.6°C for several hours, which killed four crabs. We immediately transferred the active crabs into the back-up rearing tank system; therefore, DO concentration primarily increased to approximately saturation. The increased DO concentration gradually decreased to approximately 1 mg L^−1^ in the following 5 d. pH also increased to 8.2 in the new artificial sea water, and gradually decreased. Three additional crabs died during the period between the 43rd day and the 55th day. It was possible that the death of the crabs was caused by the high temperature following the malfunction of the cooling unit. Finally, 44 of the initial 60 crabs remained (survival of crabs was 73.3%) after 84 d of cultivation when the crabs were cultivated with H_2_S feeding. On the other hand, 27 of the initial 30 crabs survived (survival of crabs was 90.0%) when the crabs were cultivated without H_2_S feeding (data not shown). The survival rate of crabs with H_2_S feeding seemed to be lower than that of crabs without H_2_S feeding. Active busy setae were observed a few days after continuous feeding of H_2_S, and their color changed from brown to cream. Furthermore, white bacterial films (biofilm) formed and increased on the surface of the tank wall (Fig. 3A) and sea sand in the feeding unit (Fig. 3B) (11 in Fig. 1A).

### Estimation of amount of epibionts on surface of setae

**Fig. 4:**
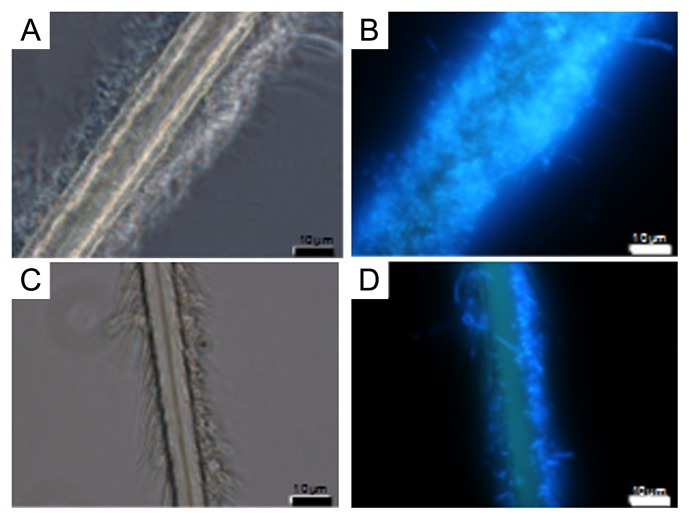
Microphotographs of setae of crabs with and without H_2_S feeding. Images of setae of crabs with feeding (A and B) and without feeding (C and D) are shown. Phase difference microscopic images (A and C) and DAPI-stained images (B and D) are shown.

**Fig. 5:**
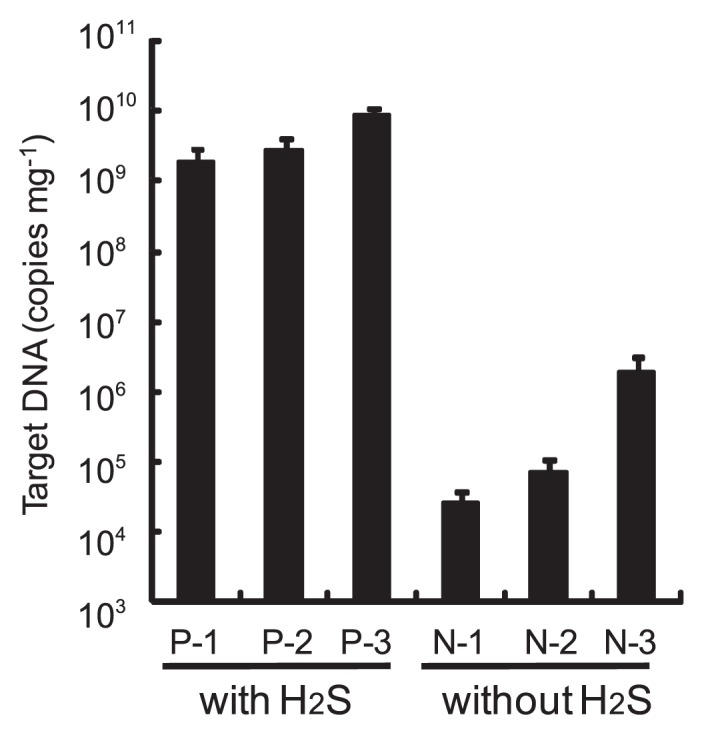
Quantification of partial 16S rDNA (341–534) using two universal primers by real-time PCR analysis. P-1, P-2, and P-3 indicate setae samples of crabs cultivated with H_2_S feeding. N-1, N-2, and N-3 indicate setae samples of crabs cultivated without the feeding. Error bars indicate standard deviations. Chain reactions were performed at least three times.

To estimate the amount of epibiotic bacteria on the surface of setae of crabs with and without H_2_S feeding after cultivation for 84 d, microscopy observations and real-time PCR analysis were performed. Microscopy images of representatives of DAPI-stained setae are shown in Fig. 3. The numbers of epibionts on setae after cultivation with H_2_S feeding (Fig. 4A and B) were obviously larger than those without H_2_S feeding (Fig. 4C and D). Interestingly, in the images of setae of crabs with feeding, spherical and cocoid epibionts were interestingly found along the filaments. The majority of epibionts on the setae of the crabs collected from the environment are filaments, as shown by previous studies (27, 30). The amount of epibionts estimated by real-time PCR analysis is shown in Fig. 5 as the copy number of target DNA (partial 16S rRNA gene) per wet weight of a sample. Three crabs from each cultivating condition were used for the analysis. The amount of epibionts of crabs with feeding reached the target 1.8×10^9^, 2.7×10^9^, and 8.2×10^9^ copies per gram of wet weight of setae, whereas those of crabs without H_2_S feeding reached 2.7×10^4^, 7.1×10^4^, and 1.9×10^6^ copies g^−1^. Although differences in the target DNA copy numbers of individual samples were observed under the same conditions, the number of copies of target DNA of epibionts of crabs with feeding was three orders of magnitude larger than that of crabs without feeding.

### Phylogenetic analysis of epibionts

**Fig. 6:**
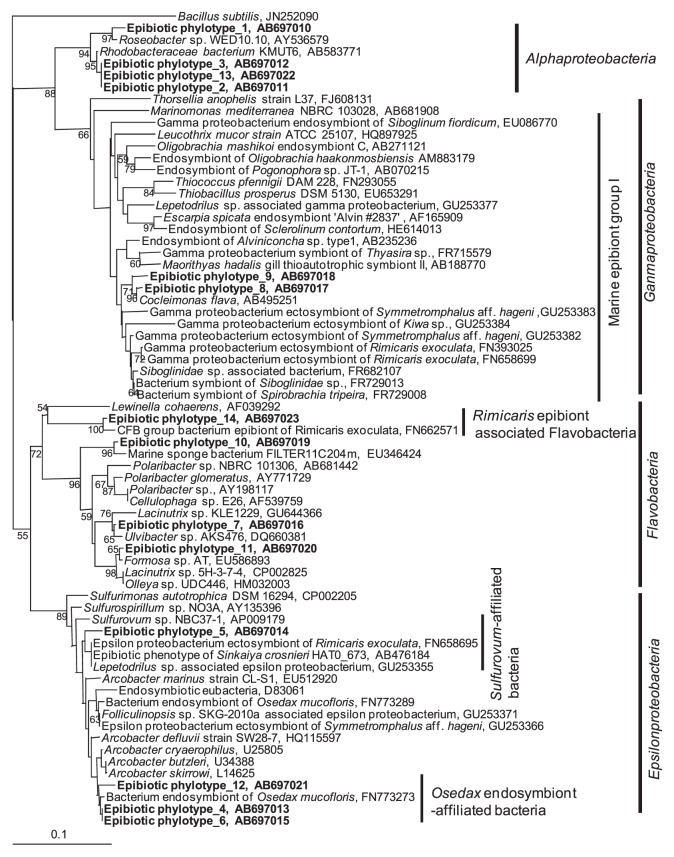
Phylogenetic tree of epibionts of *S. crosnieri* based on partial 16S rRNA gene sequences (193 nucleotides). Bootstrap values (in percent values) are based on 1,000 replicates of the neighbor-joining method and show more than 50% bootstrap support.

To determine the phylogenetic affiliation of epibionts on the surface of the setae, phylogenetic analysis was performed on the basis of the partial 16S rDNA sequences. As shown in Fig. 6, thirteen phylotypes of epibionts of the crabs cultivated with H_2_S feeding were placed in four different phyla, including *Alphaproteobacteria*, *Gammaproteobacteria*, and *Epsilonproteobacteria* and *Flavobacteria*.

One of the signature episymbiotic bacteria of *S. crosnieri*, epibiotic phylotype 5, was related to the *Sulfurovum*-affiliated bacteria group in *Epsilonproteobacteria*, which was also often detected in different *S. crosnieri* (27, 30). It was also related to ectosymbiontic clones isolated from the deep-sea limpet, *Symmetromphalus* aff. *hageni* (4), a gill endosymbiont of the hydrothermal vent gastropod, *Alviniconcha* aff. *hessleri* (22), ectosymbiont of the hydrothermal shrimp, *Rimicaris exoculata* (5), and a deep-sea snail of a *Lepetodrilus* associated *Epsilonproteobacterium* (4).

In this study, other phylotypes were also detected in *Epsilonproteobacteria*, as a bone-eating worm *Osedax mucofloris* endosymbionts-related phylotypes, namely, *Osedax mucofloris* endosymbiont-affiliated bacteria (29). There was no description of the endosymbiont in *Epsilonproteobacteria* affiliated to *Osedax mucofloris* in a recent paper (29). An *Arcobacter* sp. of the most closely related isolate is a cold-adapted mesophilic and heterotrophic bacterium (28). Species in the genera *Arcobacter* are often detected from deep sea sulfur mats near and on seeps (16). The phylotypes are thought to be deep sea sulfur oxidizers.

Other signature episymbiotic bacteria of *S. crosnieri* are well known to cluster, namely, marine epibiont group I (30), belonging to the phylum of *Gammaproteobacteria*. Epibiotic phylotype 8 and 9 are located in marine epibiont group I, although the most closely related sequence is that of *Cocleimonas flava* (GenBank Accession AB49521) isolated from the sand snail *Umbonium costatum*, which lives by burrowing into shallow sand or mud (23). Various sequences related to the symbionts of deep-sea invertebrates close to the phylotypes were located as the *Thyasira* sp. symbiont (19), *Marithyas hadalis* gill thioautotrophic symbionts (3), *Alviniconcha* sp. type I endosymbiont (22), *Siboglinum fiordicum* endosymbionts (24), *Oligobrachia haakonmosbiensis* endosymbiont (13), *Kiwa* sp. ectosymbiont (4), *Symmetromphalus* aff. *hageni* ectosymbiont (4), *Spirobrachia tripeira* symbiont (20), and *Rimicaris* sp. ectosymbiont (5), which seem to be putative sulfur-oxidizing bacteria.

Epibiotic phylotype 14 was detected in the phylum of *flavobacteria*. The most related sequence was that of an epibiont of *Rimicaris exoculata*, namely, *Rimicaris* epibiont-associated *Flavobacteria* (5). Furthermore, the other phylotypes were located near those of free-living *Alphaproteobacteria* and *Flavobacteria*. Epibiotic phylotypes 7 and 11 were related to *Formosa* sp. (14), which were detected by denaturing gradient gel electrophoresis analysis of a commercial shallow raceway marine recirculation system, and related to *Lacinutrix* sp. strain 5H-3-7-4 (8), which is a polysaccharide-degrading strain isolated from the subseafloor sediments. All of the phylotypes belonging to *Alphaproteobacteria* were related to the species of rhodobacterales, which are often isolated from marine environments as determined on the basis of GenBank information. The putative free-living bacteria were observe as spherical and cocoid by microscopy.

### Phylogenetic analysis of biofilms

**Fig. 7:**
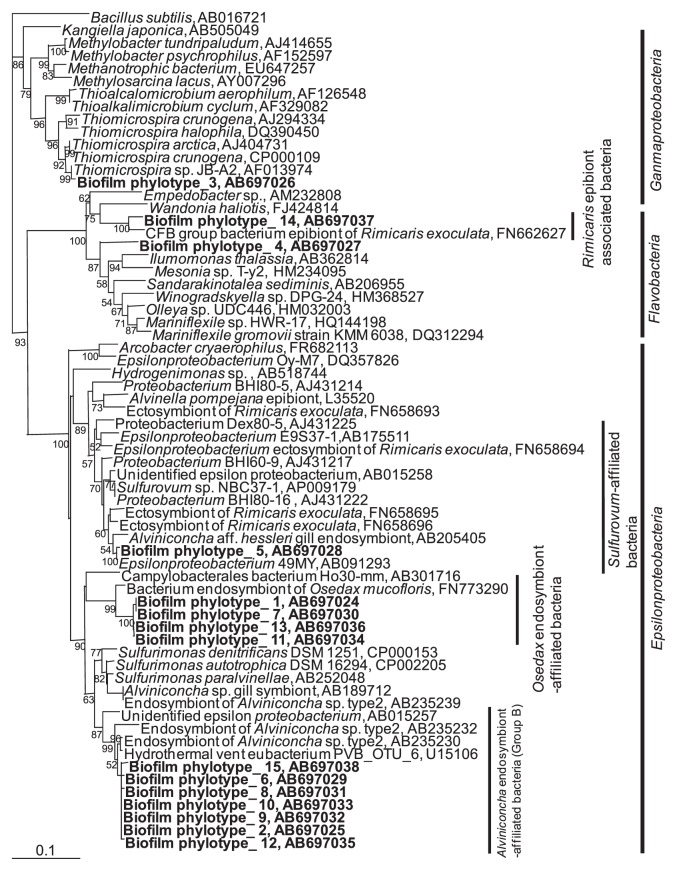
Phylogenetic tree of members of biofilm based on near-complete 16S rRNA gene sequences (1,160 nucleotides). Bootstrap values (in percent values) are based on 1,000 replicates of neighbor-joining method and show more than 50% bootstrap support.

During the cultivation experiment, white bacterial mats and films accumulated on the surfaces of the bed of sea sand in the H_2_S feeding device and tank wall (Fig. 3A and B). The DAPI-stained films were then observed by microscopy (Fig. 3C and D). The biofilms seemed to consist of bacterial consortia; therefore, phylogenetic analysis was carried out to examine the profile of the growth-stimulated bacteria in biofilm, which were collected from the tank wall 70 d after the start of cultivation. The phylogenetic tree of consortia in the biofilms is shown in Fig. 7. Fourteen bio-film phylotypes were located in *Gammaproteobacteria* and *Epsilonproteobacteria* and *Flavobacteria*; however, no phylotype belonging to *Alphaproteobacteria* was detected.

Phylotypes were categorized into the following groups: *Rimicaris* epibiont-associated *Flavobacteria*, biofilm phylotype 14; *Sulfurovum*-affiliated bacteria, biofilm phylotype 5; and *Osedax mucofloris* endosymbiont-affiliated bacteria, biofilm phylotypes 1, 7, 11, and 13. Interestingly, seven phylotypes were closely related to the sequences of endosymbionts of *Alviniconcha* sp. type 2 (22), and hydrothermal vent eubacterium PVB_OTU_6, with sequence identities of less than 96%. Phylotypes that clustered with endosymbiont sequences were detected from snails collected from the Vienna Woods site, Manus Basin, 3°9.8′S/150°16.7′E, 2510 m. The clade was named group B in a previous study (22).

Biofilm phylotype 3 of *Gammaproteobacteria* was closely related to free-living *Thiomicrospira* species with 96% sequence identity; therefore, the phylotype was derived from free-living *Thiomicrospira* sp and was different from marine epibiont group I.

## Discussion

To investigate the effects of hydrogen sulfide feeding on epibiotic communities on the surface of the deep-sea crab, *S. crosnieri*, a rearing tank with an H_2_S-supply mechanism was developed in this study for the first time. The crabs and epibionts were cultivated for 84 d using the tank system, and the structures of epibionts and bacterial mats obtained secondarily were analyzed by microscopy, real-time PCR analysis, and phylogenetic analyses.

Kawagucci *et al.* (7) reported that H_2_S was not detected near the *Bathymodious* colony in the Iheya North field; however, the quantification of H_2_S in natural environments, especially in the deep sea, is difficult in terms of accuracy because as a reducing agent, H_2_S spontaneously oxidizes during a long sampling period; therefore, there is possibility that the estimation was affected by spontaneous oxidization. This is one reason why there are few detailed reports on the effects of H_2_S on the invertebrates and microorganisms in the deep sea. We developed a simple semiconductor gas sensor and on-off control system attached to the rearing tank, and controlled H_2_S at a specific concentration, between 5 and 40 μmol L^−1^ during two distinguishable periods excluding the period when the cooling unit malfunctioned. From our cultivation experiments (Fig. 2A), H_2_S at concentrations lower than 40 μmol L^−1^ was continuously provided under a microaerobic condition of DO concentrations between 1.0 and 1.5 mg L^−1^, which corresponds to saturation between 8.12 and 12.2% at 5.2°C. These conditions stimulated the growth of deep-sea chemoautotrophs related to symbionts in this study. Based on the findings, therefore, the conditions may be representative conditions near hydrothermal vents in the deep sea, where chemoautotrophic ecosystems are located. In other words, H_2_S concentrations near the hydrothermal vents may be higher than those estimated by sampling analyses. The physical and chemical conditions for their growth, obtained from this experiment, will also be significant information for the isolation of deep-sea chemoautotrophs.

From the microscopy findings (Fig. 4) and real-time PCR analysis (Fig. 5) of the surfaces of setae, the growth of epibiotic bacteria appeared to be stimulated by a low concentration of H_2_S, as determined by the comparison between the two different cultivations with and without H_2_S feeding. Furthermore, phylogenetic analysis of epibionts (Fig. 6) showed that the rearing tank system increased not only the number of episymbionts such as *Sulfurovum-*affiliated bacteria and marine epibiont group I but also *Rimicaris-* and *Osedax*-affiliated symbiont groups along with several free-living heterotrophic bacteria. According to the results, putative sulfur-oxidizing bacteria belonging to *Sulfurovum-*affiliated bacteria and marine epibiont group I seem to operate via sulfur-oxidizing respiration by using H_2_S and its spontaneously oxidized compounds including sulfur, thiosulfate, and sulfite, in the artificial deep sea environment at a low temperature and a low DO concentration with H_2_S provided continuously. A deep-sea isolate *Sulfurovum* sp. NBC37-1, which is related to an episymbiont of *S. crosnieri*, was reported to be able to use elemental sulfur as both an electron acceptor and donor via its hydrogen-sulfur respiration and thiosulfate-oxidizing nitrate/oxygen respiration pathway under “mixing zone” conditions, where the redox condition shifted from reductive to oxidative, near a hydrothermal vent corresponding to the present artificial condition (17). *Rimicaris-*affiliated bacteria belonging to *Flavobacteria* and *Osedax*-affiliated symbiont groups belonging to *Epsilonproteobacteria* seemed to be cold-adapted mesophilic and heterotrophic bacteria. These bacteria may secondarily grow using metabolites from chemoautotrophs and the crabs.

Phylogenetic analysis of biofilms (Fig. 7) showed that the phylotypes belong to *Rimicaris* epibiont-associated *Flavobacteria*, *Sulfurovum*-affiliated bacteria, and *Osedax mucofloris* endosymbiont-affiliated bacteria, which correspond to the above results of the analysis of epibionts (Fig. 6). Growth of these bacteria would depend on chemical and physiological conditions other than gravitational pressure, and host-symbiont linkages might not necessarily be a significant factor. On the other hand, marine epibiont group I belonging to *Gammaproteobacteria*, which have been detected in epibiont phylotypes, was not detected. This finding implies that marine epibiont group I tightly bind to their host by a physiological linkage, not only because of environmental factors. Interestingly, one of the major clades of phylotypes was closely related to an endosymbiont of the snail, *Alviniconcha* sp. type II, which was collected from the Vienna Woods site, Manus Basin, whereas the Iheya North field in the Okinawa Trough is located approximately 3,700 km from the Vienna Woods site. The other related sequences that were obtained from environmental samples were also collected from the deep sea near the Hawaiian Islands. Significant growth factors for these bacteria are the chemical and physical conditions.

There are a lot of reports about the host-symbiont linkage of invertebrates through field work approaches (2–5, 13, 17, 19,20, 22, 24, 27, 29, and 30). Such approaches pose difficulty in clarifying the cause-effect relationship between growth factors and bacterial growth. In the present study, we were able to examine preliminarily the effects of H_2_S on the deep-sea crab *S. crosnieri* and its episymbionts in the laboratory, using our own novel rearing tank. Our rearing tank system will be a powerful tool for understanding the host-symbiont linkages of invertebrates inhabiting areas near deep-sea vents, particularly the bivalves, *Calyptogena* spp., which are considered to possess a strong host-symbiont linkage because of the reduced genome of the symbiont (10). Further developments of cultivation experiments are important for studies of deep-sea invertebrates and microorganisms. Further results of cultivating organisms will be described elsewhere in the future.
